# Investigating the Relationship Between Cardiac Function and Insulin Sensitivity in Horses: A Pilot Study

**DOI:** 10.3389/fvets.2022.899951

**Published:** 2022-07-08

**Authors:** Natasha J. Williams, Martin Furr, Cristobal Navas de Solis, Allison Campolo, Michael Davis, Véronique A. Lacombe

**Affiliations:** ^1^Department of Clinical Sciences, College of Veterinary Medicine, Oklahoma State University, Stillwater, OK, United States; ^2^Department of Physiological Sciences, College of Veterinary Medicine, Oklahoma State University, Stillwater, OK, United States; ^3^Department of Clinical Sciences, College of Veterinary Medicine & Biomedical Sciences, Texas A&M University, College Station, TX, United States

**Keywords:** insulin sensitivity (IS), myocardial velocities, diastolic function, systolic function, echocardiography

## Abstract

Metabolic syndrome in humans is commonly associated with cardiovascular dysfunction, including atrial fibrillation and left ventricular diastolic dysfunction. Although many differences exist between human and equine metabolic syndrome, both of these conditions share some degree of insulin resistance. The aims of this pilot study were to investigate the relationship between insulin sensitivity and cardiac function. Seven horses (five mares, two geldings, aged 17.2 ± 4.2 years, weight 524 ± 73 kg) underwent insulin-modified frequently sampled intravenous glucose tolerance testing to determine insulin sensitivity (mean 2.21 ± 0.03 × 10^−4^ L/min/mU). Standard echocardiograms were performed on each horse, including two-dimensional, M-mode, and pulse-wave tissue Doppler imaging. Pearson and Spearman correlation analyses were used to determine the association of insulin sensitivity with echocardiographic measures of cardiac function in 5 horses. Insulin sensitivity was found to be significantly correlated with peak myocardial velocity during late diastole (*r* = 0.89, *P* = 0.0419), ratio between peak myocardial velocity in early and late diastole (*r* = −0.92, *P* = 0.0263), isovolumetric relaxation time (*r* = −0.97, *P* = 0.0072), and isovolumetric contraction time (ρ = −0.90, *P* = 0.0374). These preliminary data suggest that decreased insulin sensitivity is correlated with alterations in both systolic and diastolic function, as measured with tissue Doppler imaging (TDI). Due to the small sample size of this study, the relationship between insulin sensitivity and myocardial function in horses requires further investigation.

## Introduction

Metabolic syndrome (MetS) is a collection of risk factors that compromise vascular function resulting in subclinical and synergistic damage in a variety of organs. This syndrome develops in genetically susceptible individuals as a result of chronic inappropriate dietary intake and insufficient physical activity (1), and has become increasingly prevalent within the human population (2). An individual with metabolic syndrome is defined by the National Cholesterol Education Program (NCEP) as having three or more of the following cardiovascular risk factors: central obesity (waist circumference ≥102 cm men, ≥88 cm women); increased triglycerides (≥150 mg/ml); diminished HDL cholesterol (men <40 mg/dl, women <50 mg/dl); systemic hypertension (≥130/≥85 mm Hg); and increased fasting glucose (≥110 mg/dl, i.e., insulin resistance) (1). In 2004, this NCEP definition was revised (rNCEP) by lowering the threshold for fasting glucose to ≥100 mg/dl, and including patients being treated for dyslipidemia, hyperglycemia, or systemic hypertension. In both humans and in animal models, metabolic syndrome is an established risk factor for cardiac disease, including systolic and diastolic dysfunction (3–6).

Equine metabolic syndrome (EMS) is an increasingly recognized syndrome in horses, and is defined by a collection of risk factors for endocrinopathic laminitis, with insulin dysregulation a central feature. Insulin dysregulation may also occur as a component of equine pituitary pars intermedia dysfunction, although this is not a consistent feature (7–9). Insulin dysregulation may be manifested by hyperinsulinemia; hyperglycemia; hypertriglyceridemia; and insulin resistance (10). Other risk factors include generalized or regional adiposity, although this is not a necessary feature (11). Diagnosis of EMS is based on history, clinical examination findings, and laboratory testing. Although the development of laminitis has remained the key clinical consequence of EMS, research has emerged linking EMS to cardiovascular changes, including hypertension and left ventricular hypertrophy (11, 12). Due to overlaps between MetS and EMS, including insulin dysregulation, hypertension and cardiac hypertrophy, it is plausible that EMS may also be linked to cardiac dysfunction in horses.

In this pilot study, we aimed to determine if insulin sensitivity correlates with echocardiographic parameters of cardiac function in horses. We hypothesized that decreased insulin sensitivity would be correlated with alterations in systolic and diastolic function.

## Materials and Methods

### Research Subjects

The study was performed with the approval of the Oklahoma State University (OSU) Institute for Animal Care and Use Committee (VM-18-21). Eleven horses were purchased from a local dealer based on their metabolic phenotype and two horses from the OSU teaching herd were used. Each horse was given a body condition score (13) and a cresty neck score (CNS) (14). Horses were weighed, had ages estimated based on dentition, and underwent basic physical examinations, and screening echocardiography. Blood was collected for plasma biochemistry and complete blood count, and screening for *Streptococcus equi* subsp. *equi* (nasal wash qPCR) was performed. Horses with serious underlying cardiac disease, as defined by pathologic arrhythmias or valvular disease affecting cardiac function determined by 2D echocardiography, were eliminated. During the study period, horses were maintained at pasture with free choice access to grass hay.

### Determining Insulin Sensitivity

Insulin sensitivity was determined using the insulin-modified frequently sampled intravenous glucose tolerance test (FSIGTT). Horses were stabled in pairs to minimize stress, and each horse was fasted for 10 h. Both jugular veins were clipped, aseptically prepared, and infiltrated with 2% lidocaine. An 18G 1.88-inch catheter (BD Insyte, Becton Dickinson Infusion Therapy Systems Inc., Sand, Utah) was placed in the right jugular vein for glucose and insulin administration, and a 14G 3.5-inch catheter (Extended Use MILACATH, MILA International Inc., Florence, Kentucky) was placed in the left jugular vein for sample collection. Samples were taken for basal glucose and serum insulin. The insulin-modified FSIGTT was performed as outlined by Toth et al. (15). Horses were administered 100 mg/kg dextrose IV as a 20% solution (diluted in 0.9% saline) over 5 min. Blood was collected for glucose and insulin analysis at 6, 8, 10, 12, 15, 18, 20, 23, 26, 30, 40, 50, 60, 90, 120, 150, and 180 min. Ten milliliter of waste blood was collected from the sampling catheter, followed by 10 ml of blood which was divided between fluoride oxalate (4 ml) and plain tubes (6 ml), and the catheter was then flushed with 20 ml of heparinized saline. Immediately following sample collection of the 20-min time point, 20 mU/kg regular insulin was administered to each horse through the administration catheter. Blood glucose concentration was measured in whole blood within 30 s of collection using a handheld glucometer [AlphaTRAK 2 (code 5), Zoetis Inc., Kalamazoo, Michigan. Glucose strips: lot 1074309, exp 2020/12] validated for use in horses (16), according to the manufacturer's instructions. Blood collected into plain tubes was allowed to clot, and then centrifuged at 2,500 rpm at room temperature for 10 min. Plasma and serum were separated and stored at −80°C until analysis. Insulin concentration was measured on thawed serum samples using an equine insulin ELISA (Mercodia Equine Insulin ELISA, Mercodia AB, Uppsala, Sweden. Kit lot #30096, exp 2022-02-28). Minimal model analysis (MINMOD Millenium 6.02, MinMod Inc.) was used to calculate the insulin sensitivity (S_I_, whereby a low S_I_ implies insulin insensitivity, with increasing S_I_ implying increasing insulin sensitivity) and insulin resistance index (I_R_). Insulin resistant (IR) was defined as S_I_ <1.0 × 10^−4^ L/mU/min from minimal model analysis (17–20), and the clinically used cut-off of basal insulin >20 μU/ml (20, 21).

### Echocardiography

Echocardiography was performed by an experienced operator (CNdS) using a portable ultrasonographic device (GE VIVID/I (BT12) equipped with a 1.5- to 4-MHz multifrequency sectorial transducer, with simultaneous ECG recording. All echocardiographic variables were measured using the mean of 3 non-consecutive cardiac cycles. Digitally stored recordings were used to perform all measurements offline, using electronic calipers and offline software. Standard right and left parasternal views were used to obtain 2-D, M-mode, color Doppler images, and tissue Doppler images, enabling assessment of cardiac echogenicity, size, function, valvular competence and myocardial velocities. Two-dimensional variables included pulmonary artery diameter and aortic root diameter measured from right parasternal windows (right ventricular outflow tract and left ventricular outflow tract), and left atrial size measured from a standard left parasternal (mitral valve) window (22). M-mode measurements were obtained from right parasternal short-axis views of the left ventricle at the level of the chordal attachments, and included left ventricular internal diameter during diastole and systole, interventricular septum thickness during diastole and systole, and left ventricular free wall thickness during diastole and systole. Previously reported equations (23) were used to calculate fractional shortening, mean wall thickness, relative wall thickness, and left ventricular mass. Tissue Doppler imaging (TDI) was used to obtain myocardial velocities and cardiac cycle intervals, and two-dimensional speckle tracking (2DST) was used to calculate global circumferential strain (22).

### Non-invasive Blood Pressure Measurements

Blood pressure measurements were performed using an oscillometric monitor (Cardell Veterinary Monitor 9403, CAS Medical Systems, Branford, Connecticut) with the cuff centered over the coccygeal artery around the base of the unclipped tail. A bladder width-to-tail ratio of 0.4–0.6 was used in accordance with the instructions of the monitor manufacturer. Following discard of the first measurement obtained, three further measurements were then performed and recorded, and the mean calculated (24).

### Statistical Analysis

Data were exported and analyzed using JMP Pro version 15.2.0 (SAS Institute Inc.). Data were assessed for normality through fitting a normal distribution and performing the Shapiro-Wilk test. Descriptive statistics were presented as mean ± SD for normally distributed data, and as median (IQR1–IQR3) if non-normally distributed. Pair-wise Spearman correlation analysis was used for ordinal and non-normally distributed data. Pearson correlation analysis was used for normally distributed continuous data.

## Results

### Horses

The study population consisted of a total of seven horses. Of 11 horses that were purchased, five horses met the inclusion criteria. Reasons for exclusion were: the development of colic necessitating euthanasia (*n* = 1); poor temperament 1, respiratory illness 1, pregnancy 1 or existing cardiac disease that met the previously defined exclusion criteria 2. Of the two horses excluded based on cardiac disease, one had atrial fibrillation and the other had moderate aortic regurgitation with evidence of left ventricular remodeling. Two horses were used from the OSU research herd. Horses used included five mares and two geldings, with mean (range) age, weight, cresty neck score, and body condition score of 17.7 ± 4.2 (12–23) years, 524 ± 73 (433–610) kg, 2.4 ± 1.0 (2–4), and 5.9 ± 1.2 (4.5–8), respectively. Breeds included Paint or Paint cross (*n* = 5), Thoroughbred 1 and pony 1. One horse was found to have a 2/6 systolic murmur with the PMI over the mitral valve. All nasal wash samples collected were negative for *Streptococcus equi* subsp. *equi* on qPCR, and hematology and serum biochemistry were unremarkable.

### Frequently Sampled Intravenous Glucose Tolerance Test

Based on the FSIGTT, horses were found to have a mean S_I_ of 2.21 x 10^4^ L/min/mU, and median basal fasting insulin of 7.44 mU/L (IQR 5.51–18.43) (Table 1). Using a cut-off of S_I_ <1.0 × 10^−4^ L/min/mU, one horse was classified as insulin resistant (IR) (19), whereas horses four and seven were classified as IR using basal insulin concentration cut-off of >20 μU/L (20, 21, 25). The sensitivity indices obtained from the FSIGTT of two horses were excluded from further analysis as the values obtained were considered to be outliers (with results outside the physiological range); this was presumed to be due to an error with sample handling.

**Table 1:** Basal glucose and insulin concentrations, and results from minimal model analysis of FSIGTT.

**Parameter**	**Abbreviation**	**Units**	Mean ±SD^*^	**Range**
Basal glucose	G_b_	mg/dl	114 ± 10	94–124
Basal insulin	I_b_	mU/L	7.44 [4.02, 29.3]	0.97–35.4
Acute insulin response to glucose	AIR_g_	mU/L/min	237 ± 162	69–476
Disposition index	DI		721 [317, 1,644]	14–5,884
Insulin sensitivity (× 10^−4^)	S_I_	L/min/mU	2.216	0.03–5.94
Glucose effectiveness	S_G_	min^−1^	0.014 ± 0.007	0.008–0.028
Glucose effectiveness at zero insulin (× 10^−3^)	GEZI	min^−1^	9.35 ± 8.23	0.17–25.35
HOMA_β_		mu/mM	51.5 [22.97, 174.58]	12.93–190.31
HOMA_IR_		mM.mU/L^2^	1.98 [1.25, 8.58]	0.22–11.89
R^2^		%	98.27 ± 0.78	96.78–99.17

* *Data reported as median [IQR1, IQR3] where non-normally distributed. AIR_g_, acute insulin response to glucose; DI, disposition index; FSD, fractional standard deviation; G_b_, basal glucose; GEZI, glucose effectiveness at zero insulin; HOMA_β_, β-cell function; HOMA_IR_, insulin resistance; I_b_, basal insulin; S_G_, glucose effectiveness; S_I_, insulin sensitivity*.

### Echocardiography

All horses had normal cardiac size and function determined by subjective appearence, 2D and M-mode (Table 2). Two horses were found to have mild aortic regurgitation with thickening of aortic valve or parallel fibrous band. Two other horses had mild pulmonic or mitral regurgitation. Another horse had a hyperechoic area near the cranial papillary muscle, which was suspected to be a fibrous or fibro fatty infiltrate. Data for ejection time was missing for one horse.

**Table 2:** Echocardiographic measurements (*n* = 7). GSC data are presented as median (IQR1-IQR3). due to non-normal distribution.

	**Parameter**	**Mean ±SD**	**Range**	**Units**
**2D**	PAD	6.5 ± 0.4	6.0–7.1	cm
	AoD	6.5 ± 0.5	6.5–8.0	cm
	LADmax	10.7 ± 0.5	9.8–11.3	cm
	LAAmax	81.5 ± 9.4	67.1–91.4	cm^2^
	LAAa	70.1 ± 4.3	62.4–74.2	cm^2^
	LAAmin	47.4 ± 4.0	40.8–50.9	cm^2^
	Ac LA FAC	32.3 ± 5.5	26.2–43.3	%
	Pa LA FAC	13.1 ± 9.8	0.1–25.5	%
	LA-RI_(area)_	73.1 ± 26.3	44.2–124.0	%
	LVIAd	126.7 ± 12.7	103.6–142.8	cm^2^
	LVIAs	54.0 ± 6.2	43.1–63.0	cm^2^
	SV	687 ± 111	518–822	ml
	SV mod	629 ± 110	450–765	ml
	CO	28.14± 8.85	17.34–43.16	L/min
	CO mod	25.77 ± 8.30	15.08–40.18	L/min
	R-R	1,576 ± 236	1,135–1,863	ms
	LADLmax	11.6 ± 0.8	10.6–12.5	cm
**Mmode**	LVIDs	6.1 ± 0.4	5.6–6.7	cm
	LVIDd	10.6 ± 0.6	9.5–11.4	cm
	IVSs	4.4 ± 0.2	3.9–4.6	cm
	IVSd	2.7 ± 0.2	2.4–3.0	cm
	FS	43 ± 4	37–48	%
	LVFWs	3.6 ± 0.4	2.9–4.0	cm
	LVFWd	2.0 ± 0.2	1.8–2.3	cm
	RWT	0.46 ± 0.03	0.40–0.48	
	MWT	2.40 ± 0.14	2.20–2.55	cm
	LVMass	2,591 ± 366	1,983–3,009	g
	EPSS	4.1 ± 1.9	2–7	mm
	PEP	68 ± 19	42–101	ms
	ET	467 ± 31	274–501	ms
	RVIDd	3.1 ± 0.2	2.8–3.3	cm
	RVIDs	2.8 ± 0.7	2.1–4.2	cm
**TDI**	Variables of LV systolic function			
	S_1_	6.9 ± 1.3	5.0–9.0	cm/s
	S_m_	11.0 ± 3.1	6.0–14.0	cm/s
	PEP_m_	92 ± 7	31–101	ms
	IVCT_m_	118.3 ± 25.4	85–161	ms
	ET_m_	435 ± 28	392–471	ms
	Variables of LV diastolic function			
	E_1_	7.9 ± 0.6	7.0–9.0	cm/s
	E_m_	22.9 ± 2.3	21.0–27.0	cm/s
	E_m_/A_m_	2.30 ± 0.45	1.50–3.00	
	IVRT_m_	64.4 ± 17.7	42–87	ms
	Variables of active LA function			
	A_m_	10.3 ± 2.2	7.0–14.0	cm/s
	LA velocity	8.4 ± 1.4	7.0–11.0	cm/s
**2DST**	GSC	−16.5 (−17.1 to −15.0)	−17.5 to −12.9	%

*2DST, two-dimensional speckle tracking echocardiography; a, at maximal atrial contraction; A, late diastolic peak measured by TDI, following the ECG P wave; Ac, active; A_m_, peak late-diastolic LV wall motion velocity at the time of atrial contraction; AoD, aortic (sinus) diameter; d, at the end of ventricular diastole; E, early diastolic peak measured by TDI, following the ECG R wave; E_1_, peak LV wall motion velocity during the phase of isovolumetric relaxation; ε_CC_, circumferential strain; ε_LL_, longitudinal strain; E_m_, early-diastolic peak LV wall motion velocity during the phase of rapid ventricular filling; E_m_/A_m_, ratio between peak radial wall motion velocities during early (E_m_) and late (A_m_) diastole; EPSS, e-point septal separation; ET, left ventricular ejection time; ET_m_, ejection time (measured with TDI); FAC, fractional area change; FS, fractional shortening; GSC, global circumferential strain; IVCT_m_, isovolumetric contraction time; IVRT_m_, isovolumetric relaxation time; IVS, interventricular septum thickness; L, left (from left); LA, left atrium; LAA, left atrial area; LAAmax, maximum left atrial area, (determined 1 frame before opening of the mitral valve); LAAa, left atrial area at onset of active contraction (determined beginning of p wave); LAAmin, minimum LA area (determined at closure of the mitral valve); LAD, LA internal diameter; LA-RI_(area)_, LA reservoir index determined by area; LA veloc, LA velocity; LV, left ventricle; LVEF, LV ejection fraction; LVFW, left ventricular free wall thickness; LVMass, left ventricular mass (LVM); LVIA, LV internal area; LVIDd, LV internal diameter in diastole; LVIDs, LV internal diameter in systole; MetS, human metabolic syndrome; mod, modified; MWT, mean wall thickness; p, at the onset of the P wave; Pa, passive; PAD, pulmonary artery diameter; PEP, left ventricular pre-ejection period; PEP_m_, pre-ejection period (measured with TDI); PW, pulsed-wave; RA, right atrium; R-R, time elapsed between two successive R-waves of the QRS signal on the electrocardiogram; RV, right ventricle; RVIDd, RV internal diameter in diastole; RVIDs, RV internal diameter in systole; RWT, relative wall thickness; s, at the end of ventricular systole; SV, stroke volume*.

### Non-invasive Blood Pressure Measurements

Horses had mean (±SD) systolic, diastolic and mean arterial blood pressures of 121 ± 13, 70 ± 17, and 87 ± 14 mm Hg, respectively. Non-invasive blood pressure measurement was not collected in three horses, in part due to poor temperament.

### Correlations With Insulin Sensitivity Parameters

**Figure 1:**
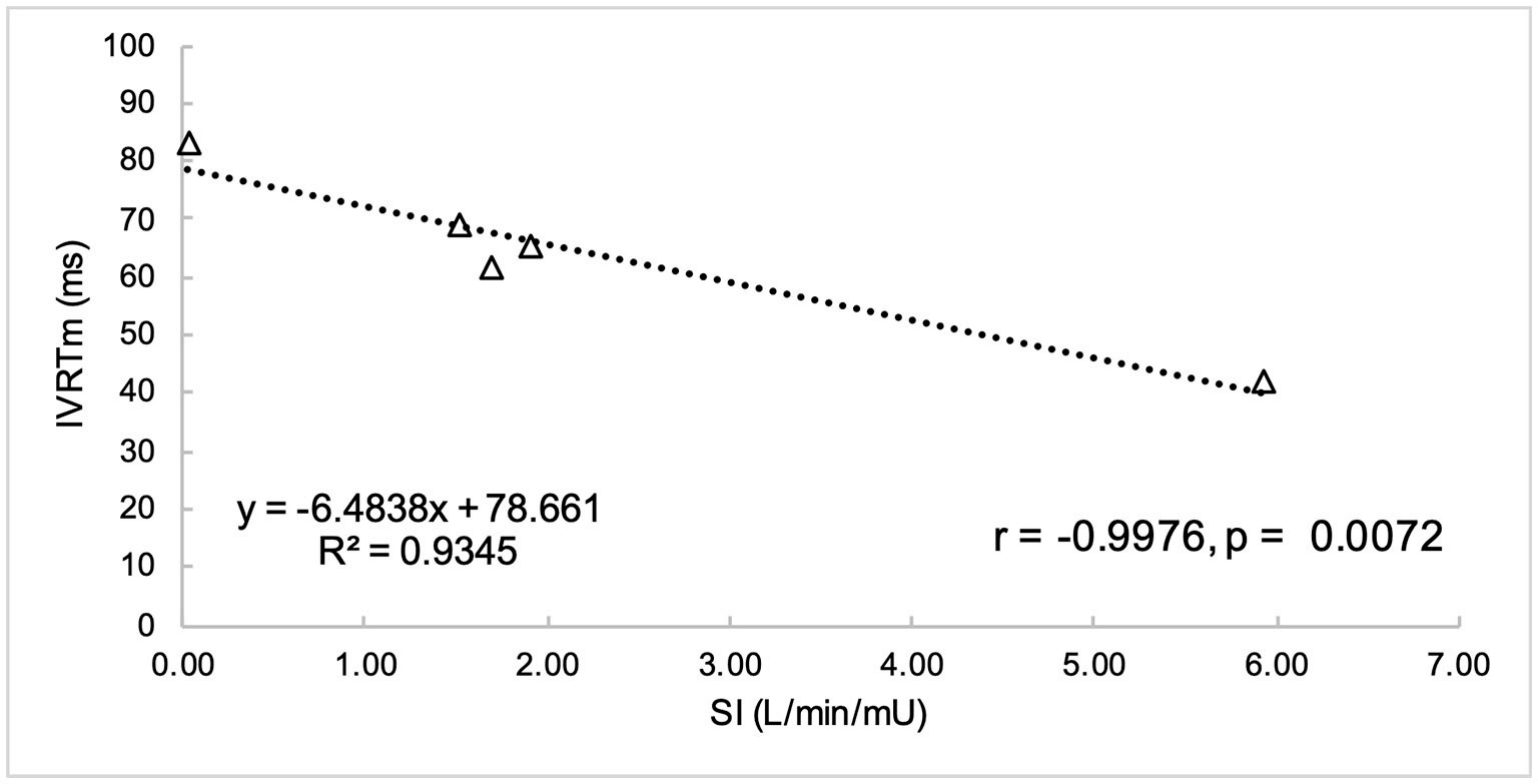
Insulin sensitivity (S_I_) was negatively correlated with isovolumetric relaxation time (IVRT_m_).

**Figure 2:**
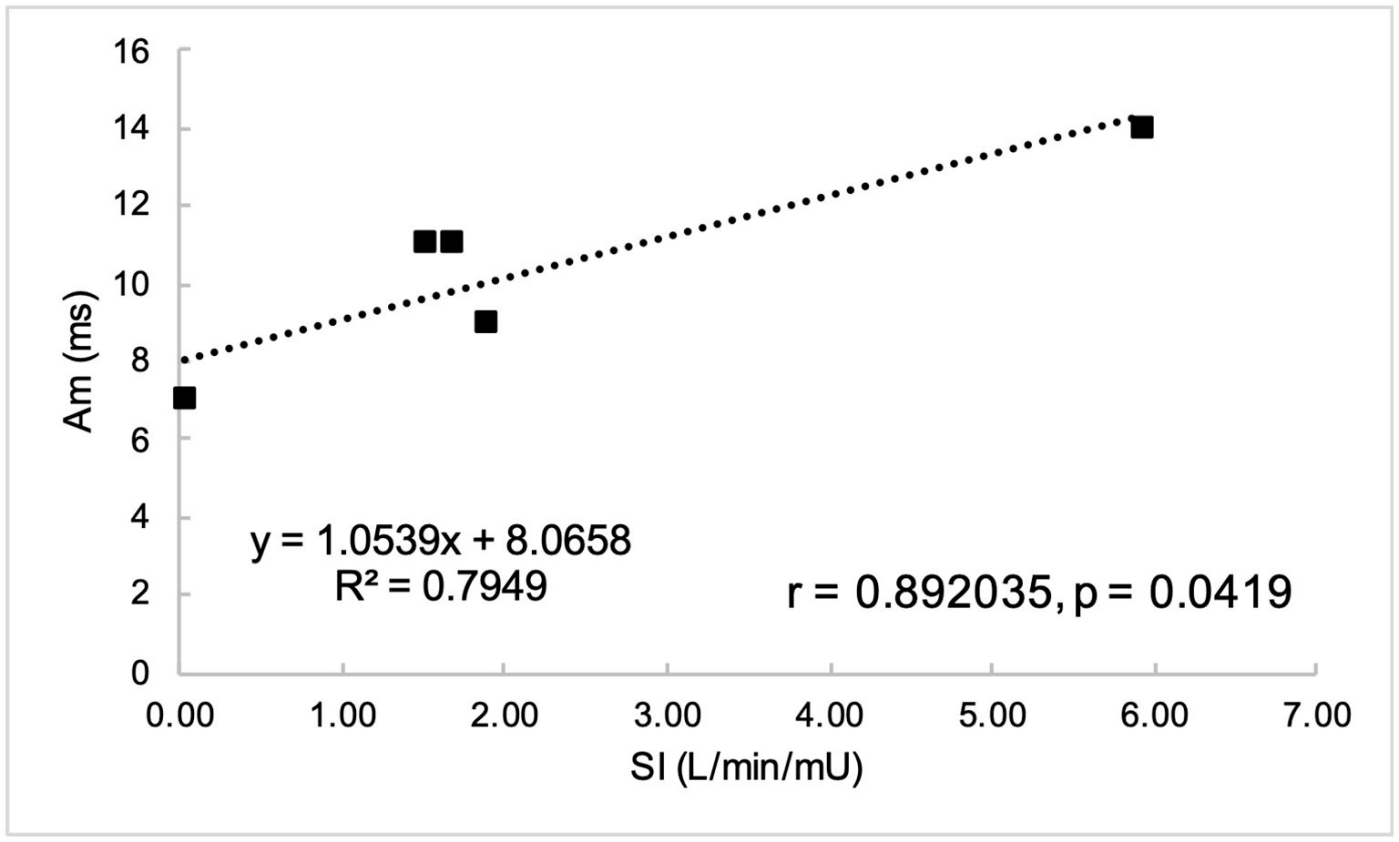
Peak myocardial velocity during active atrial contraction in late diastole (A_m_) was positively correlated with insulin sensitivity (S_I_).

**Figure 3:**
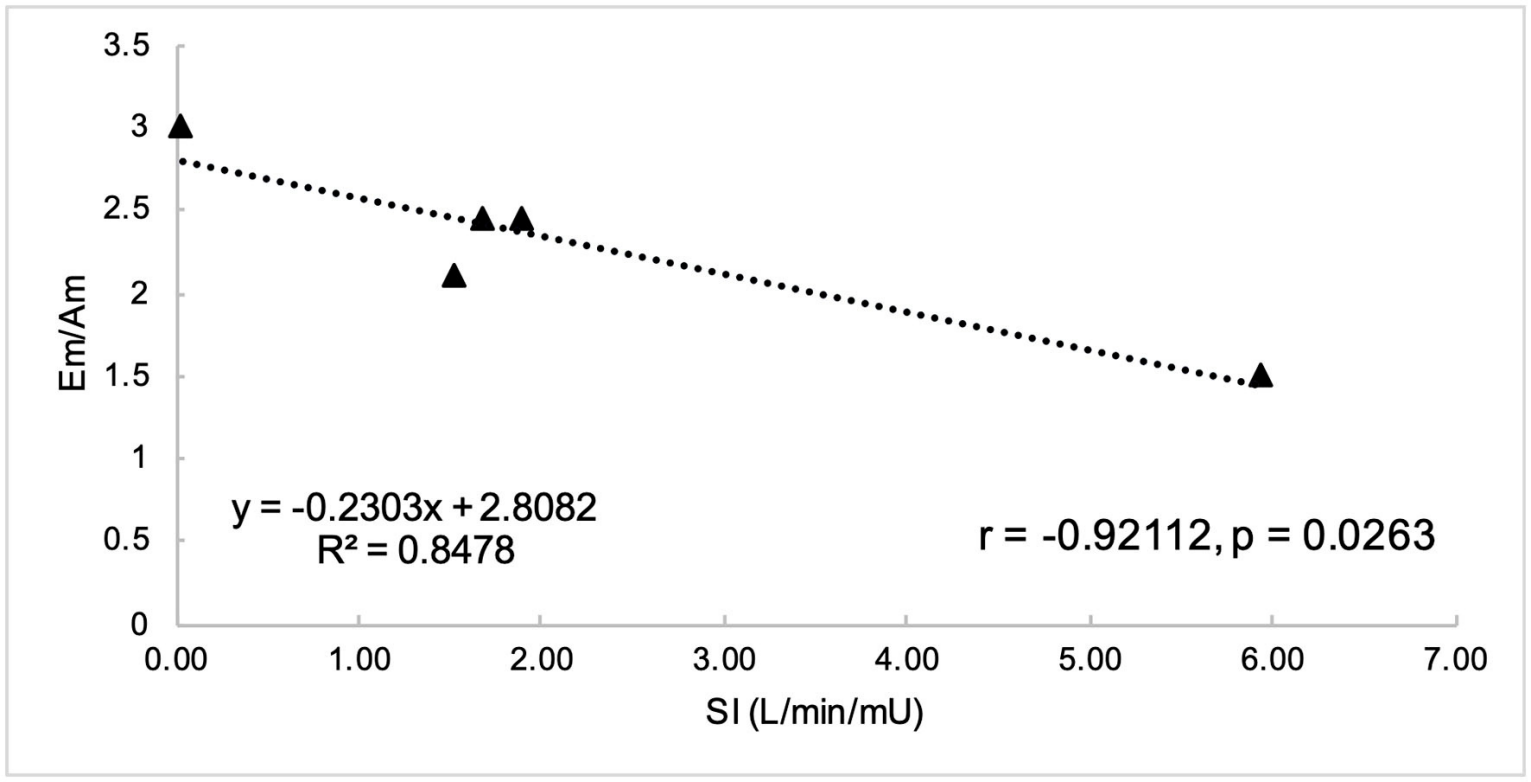
Insulin sensitivity (S_I_) was negatively correlated with ratio of myocardial velocity during early (E_m_) and late (A_m_) diastole.

**Figure 4:**
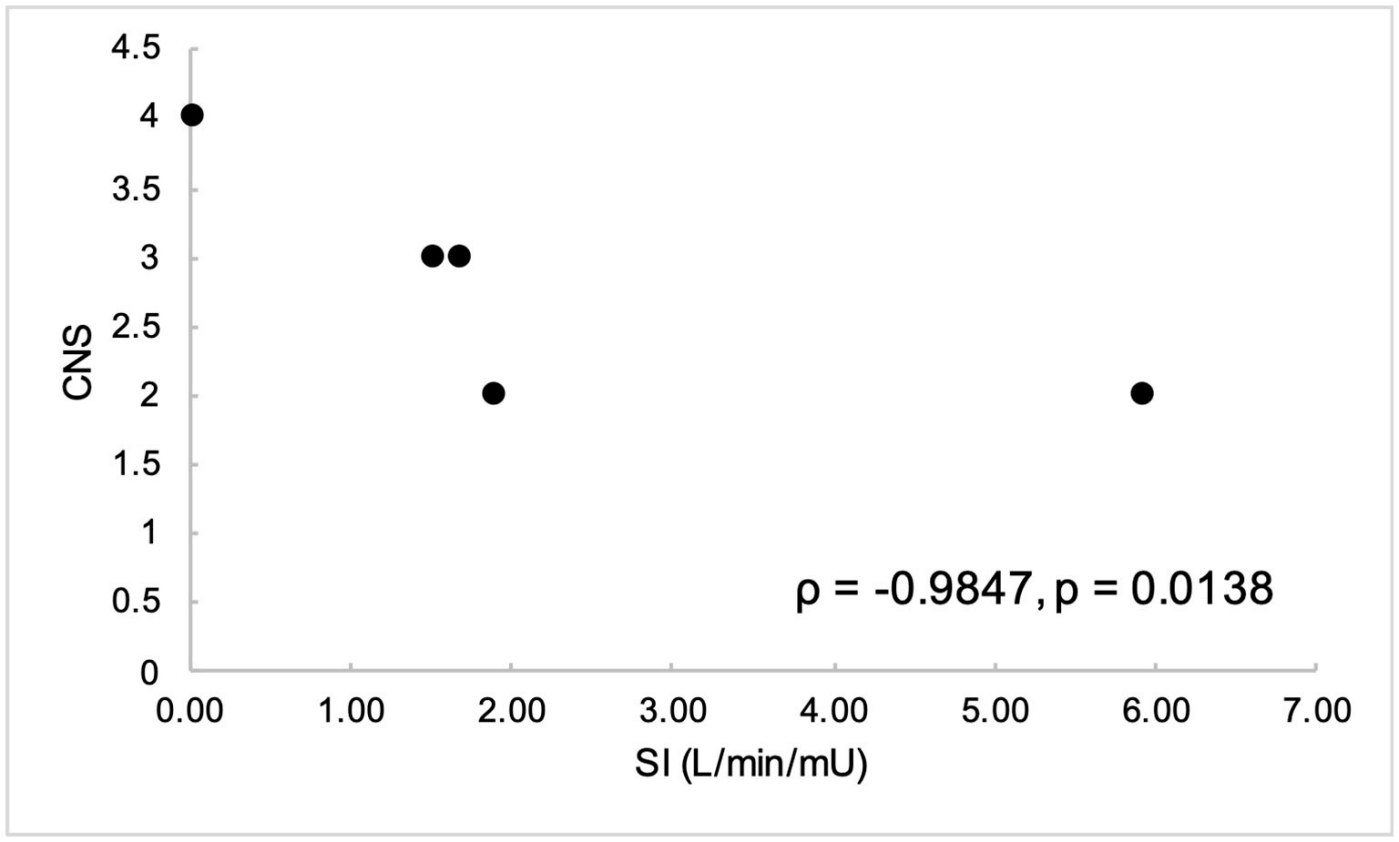
Cresty neck score (CNS) was negatively correlated with insulin sensitivity (S_I_).

**Figure 5:**
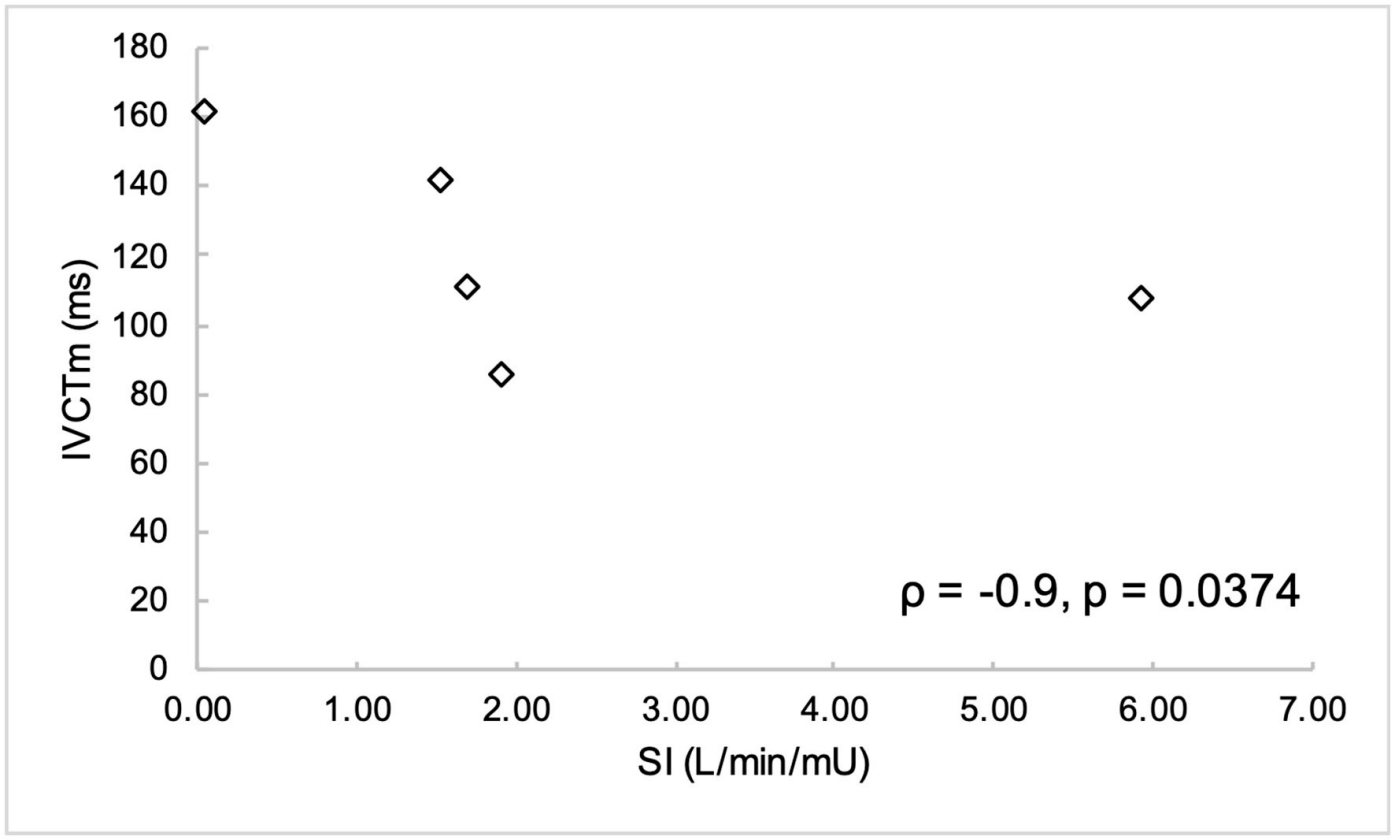
Isovolumetric contraction time (IVCT_m_) was negatively correlated with insulin sensitivity (S_I_).

Non-normally distributed parameters included HOMA_IR_, HOMA_β_, disposition index, basal insulin, and global circumferential strain. These parameters and ordinal data were analyzed using Spearman correlation. Remaining variables were normally distributed and were analyzed using Pearson correlation. Parameters of insulin dysregulation (S_I_, HOMA_β_, HOMA_IR_, etc) were compared pairwise with echocardiographic parameters of cardiac function. Insulin sensitivity was associated with myocardial function, as evidenced by a positive correlation with peak velocity of the myocardium during the period of diastole corresponding with active atrial contraction (A_m_, *r* = 0.89, *P* = 0.0419), and a negative correlation with isovolumetric relaxation time (IVRT_m_, *r* = −0.97, *P* = 0.0072) (Figures 1, 2). Insulin sensitivity also correlated with increases in the ratio of myocardial velocities during early and late diastole (E_m_/A_m_, *r* = 0.92, *P* = 0.0263), (Figure 3), and negatively with isovolumetric contraction time (IVCT_m_, ρ = −0.90, *P* = 0.0374, Figure 4), as well as cresty neck score (CNS, ρ = −0.95, *P* = 0.0138, Figure 5). No significant correlations were found between decreases in insulin sensitivity and blood pressure, nor measures of ventricular size.

## Discussion

### Insulin Sensitivity

The insulin sensitivity index (S_I_) reflects insulin-dependent glucose clearance. In the current study, only one horse was classified was insulin resistant (S_I_ = 0.03 L/min/mU) using the cut-off of S_I_ < 1.0 × 10^−4^ L/min/mU proposed by Burns et al. (19), with five horses classified as insulin sensitive (S_I_ ranges 1.515 to 5.94 L/min/mU), based on the proposed cut-off of S_I_ < 1.0 x 10^−4^ L/min/mU (19). In the study by Burns et al. (19), the authors performed minimal model analysis on data obtained from a FSIGTT. Notable differences exist between the methodology of that study and the study reported here, including differences in their FSIGTT protocol (500 mg/kg dextrose IV) and insulin dosage and measurement (100 mU/kg, radioimmunoassay). Due to the various methods for measuring insulin (26–30) and variations on FSGITT protocols (15, 31, 32), comparing results between studies presents a challenge. The intention of the study reported here was to obtain quantitative measurements of degree of insulin sensitivity in order to compare with various cardiac parameters, and this goal was achieved.

Using a fasting insulin cut-off of >9.5 of μU/ml (25), two horses in this study would have been classified as insulin resistant. Compared with the gold standard of euglycemic hyperinsulinemic clamp, fasting insulin, as measured with the Mercodia equine insulin ELISA, has been reported to have a sensitivity and specificity of 91% and 85%, respectively, for diagnosing insulin resistance using the cut-off of >9.5 of μU/ml (25). Many different cut-offs have been described for categorizing horses as insulin resistant, and it is important to note that cut-offs vary depending on the laboratory and assay used for measurement of insulin. The utility of using fasting insulin for the diagnosis of insulin resistance continues to be debated, as fasting alone may put horses into an insulin-resistant state (33).

### Myocardial Structure

In this study, there was no significant correlation of insulin sensitivity with relative wall thickness (RWT), mean wall thickness (MWT) or left ventricular (LV) mass, which are indices reflective of ventricular remodeling. This is in contrast to studies in ponies with EMS (12, 34), which found increases in MWT and RWT without increases in LV mass, indicative of concentric remodeling. In humans, MetS is associated with increased RWT (35, 36), indicating concentric remodeling. An increased LV mass has also been reported in humans with MetS (35, 37), however this finding was found to be more strongly associated with blood pressure (35). The study by Heliczer et al. (12) found that MWT and RWT were increased in Shetland ponies with EMS compared to controls, however for all ponies, only RWT was increased in the cases with EMS, which may imply an existence of breed differences in response to metabolic syndrome. Duration of insulin resistance may also be an important factor in the development of gross cardiac remodeling, which requires further investigation.

### Myocardial Function

Our study found significant correlations between insulin sensitivity and indices of both diastolic and systolic function. To the author's knowledge, this is the first study to investigate the relationship between insulin sensitivity in horses and its effect on myocardial function as evaluated with TDI and 2DST. Comparing systolic and diastolic functional indices between humans and horses is challenging due to the associated difficulties in reliably obtaining certain echocardiographic measurements in horses. In humans, pulsed-wave (PW) Doppler-derived transmitral flow velocities (E wave, A wave, E/A ratio, E-wave deceleration time, and deceleration slope) are commonly used in combination with TDI-derived wall motion velocities (E_m_, A_m_, E_m_/A_m_ ratio) for the assessment of diastolic left ventricular and filling pressures (38). In adult horses, PW Doppler recordings of transmitral flow velocities are difficult to obtain and relatively unreliable due to challenges associated with sample positioning (22, 39). The use of TDI-derived wall motion velocities may provide a more accurate assessment of left ventricular diastolic function in horses than PW Doppler.

#### Systolic Function

Decreases in insulin sensitivity were associated with increases in isovolumetric contraction time (IVCT_m_, ρ = −0.94, *P* = 0.04), suggesting impaired systolic contractile function. Isovolumetric contraction time represents the time period in early systole following closure of the atrioventricular valves, but before enough intraventricular pressure has been generated to open the semilunar valves. Systolic dysfunction has also been documented in humans with MetS, as evidenced by decreases in ε, strain rate and S_m_ (6, 37, 40). Although IVCT_m_ has been found to be significantly increased in horses with moderate to severe mitral valve regurgitation (22), only one horse in the present study had mitral regurgitation, which was considered to be mild.

A decrease in pre-load can also increase pre-ejection period and decrease LV ejection time, causing decreases in systolic time intervals (22). Decreases in pre-load may be reflected by decreases in LADmax, LAAmax, and LVIDd (22). Higher heart rates also result in decreased pre-load, as higher heart rates result in shorter filling times for both the left atrium and LV (22). None of the horses in this study had increased heart rates, and no correlations were found between parameters of pre-load and systolic time intervals, thus the observed systolic time intervals are unlikely to have been affected by pre-load.

Our study did not find a correlation between insulin sensitivity and global circumferential strain (ε). When strain is used as index of systolic function, shortening is indicated by negative strain. Thus, the more negative the strain, the better the LV systolic function. In humans, MetS is significantly associated with less circumferential myocardial shortening as indicated by less negative LV circumferential strain (ε_CC_) (37), and less longitudinal myocardial shortening as indicated by less negative LV longitudinal strain (ε_LL_) (37). The magnitude of changes in mean systolic strain and peak systolic strain rate also increased with number of components of MetS (6, 40). Other measures of systolic function found to be significantly decreased in humans with MetS include TDI-derived systolic septal myocardial velocity (a measure of longitudinal myocardial contractility) (36), and peak myocardial systolic velocity (S_m_,) (6, 36). The lack of association found between insulin sensitivity and cardiac strain or peak systolic myocardial velocity in our study may represent species differences in cardiac metabolism. However, it is also important to note our study was comprised of a very small sample size, and only one horse was classified as insulin resistant, thus further studies with larger numbers of both insulin-sensitive and insulin-resistant horses are needed to investigate this relationship.

#### Diastolic Function

In our study, decreases in insulin sensitivity were associated with diastolic dysfunction, as evidenced by a decrease in myocardial velocity during active atrial contraction (A_m_, *r* = 0.89, *P* = 0.04), and an increase in isovolumetric relaxation time (IVRT_m_, *r* = −0.9976, *P* = 0.0072) with decreases in S_I_. Isovolumetric relaxation time represents the time from the end of systole following closure of the semilunar valves and prior to opening of the atrioventricular valves, and is a sensitive measure of diastolic function. An increase in this time is reflective of a delay in myocardial relaxation, and that more time is required for enough pressure change to occur within the ventricle to induce opening of the mitral valve. Delays in myocardial relaxation may occur with catecholamine stimulation induced increases in afterload, and with aging-associated fibrosis of the ventricle (38). Tachycardia may also decrease the time available for myocardial relaxation. MetS in humans has been associated with an increase in IVRT_m_ (36), in addition to decreased peak early diastolic strain rate (6, 40).

This study found decreases in insulin sensitivity were associated with increases in the E_m_/A_m_ ratio, in contrast to the decreased E_m_/A_m_ ratio as has been found in humans with MetS (35).

The current study found decreases in insulin sensitivity were associated with decreases in peak radial wall motion velocity in late diastole (A_m_), suggesting atrial dysfunction. In humans, MetS is associated with early diastolic dysfunction, as evidenced by reduced peak radial wall motion velocity in early diastole (E_m_) (6, 35, 36), with resultant decrease in the E_m_/A_m_ ratio (35). TDI-derived early diastolic myocardial velocities were significantly lower in both MetS and pre-MetS humans than in controls, suggesting progressive impairment in LV relaxation as the number of MetS criteria increase (36). This difference may represent species differences with respect to the metabolism of the atrial myocardium in the presence of insulin dysregulation, and requires further investigation.

A marked decrease in E_m_ and E_m_/A_m_ ratio has been postulated to represent a hallmark of LV diastolic dysfunction in horses with myocardial disease (22). In a previous study by Koenig et al. (22) horses with myocardial disease showed a marked decrease in E_m_ with a non-significant increase in A_m_ velocity and a resulting significant decrease in E_m_/A_m_ ratio, suggesting impaired ventricular relaxation with a compensatory increase in left atrial booster pump function in an attempt to maintain cardiac output (22). These findings are in contrast to the current study (decreased A_m_ and increased E_m_/A_m_ ratio), and it is important to note that the horses in the Koenig et al. study had significant myocardial disease. It is likely that different areas of the myocardium are affected by insulin dysregulation, and it is difficult to make comparisons as the etiology of myocardial disease in the aforementioned study was likely heterogenous and not confirmed.

In humans, myocardial systolic and diastolic function have been found to decrease with age, leading to decreased S_m_ and E_m_, and increased A_m_ velocities (41). In a population of healthy warmblood horses, age was not found to significantly influence PW TDI variables (22), however the mean age in that study was 12 ± 4 years. The mean age of horses in the current study was older at 17 ± 4 years, and this may have confounded the differences in myocardial velocities. The influence of age on E_m_, A_m_ and other TDI variables requires further investigation.

### Other Findings

In our study, decreases in insulin sensitivity were not correlated with blood pressure. Studies in ponies have reported hypertension associated with metabolic syndrome (11, 12, 34), preceding left ventricular hypertrophy (34). The study by Heliczer et al. (12) found that in Shetland ponies, but not all ponies, those with EMS had significant increases in systolic and mean arterial blood pressures compared to controls. It is important to note that in the study by Heliczer et al., cases were designated as having EMS based on history of laminitis, body condition score (≥7/9), cresty neck score (≥3/5), and abnormal oral sugar test results (using a dosage of 0.15 ml/kg corn syrup). As the oral sugar test lacks sensitivity, but is very specific, for the diagnosis of insulin resistance (20), the ponies used in the aforementioned study are likely to have had much greater degrees of insulin resistance compared to the horses in our study population, which may account for differences seen. Human patients with MetS have also been found to have significant increases in systolic and diastolic blood pressures, concurrently with significant linear increases in ventricular (septal and posterior) wall thickness and LV mass index compared with controls (40).

Correlation was found between cresty neck score (CNS) and insulin sensitivity index (ρ = −0.95, *P* = 0.0138). In a previous study, CNS was found to moderately correlate with insulin, glucose and triglyceride concentrations in both horses and ponies (14). However, to the author's knowledge, direct correlation between CNS and S_I_ *per se* has not been yet been reported.

## Limitations

This study had several limitations. The small sample size in this pilot study limits its statistical power–as such the correlations obtained must be interpreted with caution. In order to gain confidence that any correlation obtained within the study is an accurate reflection of correlation within the equine population, this study needs to be repeated with a much larger sample size. In addition to a small sample size, only one horse in our study met the criteria for EMS, which limited our ability to investigate the relationship between cardiac function and more marked degrees of insulin resistance.

Additionally, echocardiographic measurements were only performed at one time point, which may have resulted in some variability in measurements going undetected. Another limitation was that inherent to the Doppler technique itself, as velocity measurements are affected by total heart motion and by the insonation angle between the ultrasonographic beam and wall motion. Future work should include repeating this study with a larger sample size and including horses both with and without EMS.

## Conclusion

In both humans with metabolic disease and horses with documented cardiac disease, the use of myocardial tissue velocities has enabled the detection of myocardial dysfunction prior to the occurrence of changes in more traditional measures of cardiac dysfunction (6, 42–44). In the current pilot study analyzing the results of five horses, equine insulin resistance was found to negatively impact both systolic and diastolic function, as measured with tissue Doppler imaging. The results of this study must be interpreted with caution due to the small sample size, and the relationship between insulin sensitivity and subclinical ventricular dysfunction in horses requires further investigation. Therefore, while this study adds to the body of evidence that EMS has a cardiovascular component, including diastolic dysfunction, further studies are needed to define this component.

## Data Availability Statement

The raw data supporting the conclusions of this article will be made available by the authors, upon justified request.

## Ethics Statement

The animal study was reviewed and approved by Oklahoma State University (OSU) Institute for Animal Care and Use Committee (VM-18-21).

## Author Contributions

VL and MD conceived and designed the study. All authors contributed to data collection and/or analysis and the writing and/or editing of the manuscript.

## Funding

This study was provided by awards from the Oklahoma State University College of Veterinary Medicine Research Advisory Committee (VL) and the Oklahoma State University Vice President of Research (VL and MD).

## Conflict of Interest

The authors declare that the research was conducted in the absence of any commercial or financial relationships that could be construed as a potential conflict of interest.

## Publisher's Note

All claims expressed in this article are solely those of the authors and do not necessarily represent those of their affiliated organizations, or those of the publisher, the editors and the reviewers. Any product that may be evaluated in this article, or claim that may be made by its manufacturer, is not guaranteed or endorsed by the publisher.

## Glossary

2DST: two-dimensional speckle tracking echocardiography
A: late diastolic peak measured by tissue Doppler imaging, following the ECG P wave
AIRg: acute insulin response to glucose
A_m_: peak late-diastolic left ventricular wall motion velocity at the time of atrial contraction
CNS: cresty neck score
DI: disposition index
E: early diastolic peak measured by tissue Doppler imaging, following the ECG R wave
E_1_: peak left ventricular wall motion velocity during the phase of isovolumetric relaxation
ε_CC_: circumferential strain
ECG: electrocardiogram
ε_LL_: longitudinal strain
E_m_: early-diastolic peak left ventricular wall motion velocity during the phase of rapid ventricular filling
E_m_/A_m_: ratio between peak radial wall motion velocities during early (E_m_) and late (A_m_) diastole
EMS: equine metabolic syndrome
ET: ejection time
FS: fractional shortening
FSIGTT: frequently sampled intravenous glucose tolerance test
G_b_: basal glucose
GEZI: glucose effectiveness at zero insulin
GSC: global circumferential strain
HOMA_β_: homeostatic model assessment of β-function
HOMA_IR_: homeostatic model assessment of insulin resistance
HR: heart rate
I_b_: basal insulin
ID: insulin dysregulation
IR: insulin resistance
IVCT_m_: isovolumetric contraction time
IVRT_m_: isovolumetric relaxation time
IVS: interventricular septum thickness
L: left (from left)
LA: left atrium
LAA: left atrial area
LAAmax: maximum left atrial area
LAAa: left atrial area at onset of active contraction (determined beginning of p wave)
LAAmin: minimum left atrial area (determined at closure of the mitral valve)
LAD: left atrial internal diameter
LA-RI_(area)_: left atrial reservoir index determined by area
LA veloc: left atrial velocity
LV: left ventricle
LVEF: left ventricular ejection fraction
LVFW: left ventricular free wall thickness
LVMass: left ventricular mass (LVM)
LVIA: left ventricular internal area
LVIDd: left ventricular internal diameter in diastole
LVIDs: left ventricular internal diameter in systole
MetS: human metabolic syndrome
MWT: mean wall thickness
NCEP: National Cholesterol Education Program
OSU: Oklahoma State University
p: at the onset of the P wave
PEP_m_: pre-ejection period (measured with tissue Doppler imaging)
PW: pulsed-wave
rNCEP: revised National Cholesterol Education Program
R-R interval: time elapsed between two successive R-waves of the QRS signal on the electrocardiogram
RV: right ventricle
RWT: relative wall thickness
s: at the end of ventricular systole
S_1_: peak left ventricular wall motion velocity during the phase of isovolumetric contraction
S_G_: glucose effectiveness
S_I_: insulin sensitivity index
S_m_: positive systolic wave
SR: strain rate
TDI: tissue Doppler imaging.
